# Glutamate Transporter GLT1 Expression in Alzheimer Disease and Dementia With Lewy Bodies

**DOI:** 10.3389/fnagi.2018.00122

**Published:** 2018-04-26

**Authors:** Paula Garcia-Esparcia, Daniela Diaz-Lucena, Marina Ainciburu, Benjamin Torrejón-Escribano, Margarita Carmona, Franc Llorens, Isidro Ferrer

**Affiliations:** ^1^Department of Pathology and Experimental Therapeutics, University of Barcelona, Hospitalet de Llobregat, Barcelona, Spain; ^2^CIBERNED (Biomedical Network Research Centre of Neurodegenerative Diseases), Institute of Health Carlos III, Madrid, Spain; ^3^Biology Unit, Scientific and Technical Services, University of Barcelona, Hospitalet de Llobregat, Barcelona, Spain; ^4^Bellvitge Biomedical Research Institute, l’Hospitalet de Llobregat (IDIBELL), Barcelona, Spain; ^5^Senior Consultant Service of Pathology, Bellvitge University Hospital, IDIBELL, Hospitalet de Llobregat, Barcelona, Spain; ^6^Institute of Neurosciences, University of Barcelona, Barcelona, Spain

**Keywords:** Alzheimer disease, dementia with Lewy bodies, glutamate transporter 1, EAAT2, vGLUT1, glial fibrillary acidic protein, ALDH1L1

## Abstract

Glutamate transporter solute carrier family 1, member 2 (GLT1/EAAT2), a major modulator of glutamate homeostasis in astrocytes, is assessed in post-mortem human brain samples of frontal cortex area 8 in advanced stages of Alzheimer disease (AD) and terminal stages of dementia with Lewy bodies (DLB) in order to gain understanding of astrogliopathy in diseases manifested by dementia. Glial fibrillary acidic protein (GFAP) mRNA expression is significantly increased in AD but not in DLB, whereas *GLT1*, vesicular glutamate transporter 1 (*vGLUT1*) and aldehyde dehydrogenase 1 family member 1 (*ALDH1L1*) are not modified in AD and DLB when compared with controls. GLT1 protein levels are not altered in AD and DLB but GFAP and ALDH1L1 are significantly increased in AD, and GFAP in DLB. As a result, a non-significant decrease in the ratio between GLT1 and GFAP, and between GLT1 and ALDH1L1, is found in both AD and DLB. Double-labeling immunofluorescence and confocal microscopy revealed no visible reduction of GLT1 immunoreactivity in relation to β-amyloid plaques in AD. These data suggest a subtle imbalance between GLT1, and GFAP and ALDH1L1 expression, with limited consequences in glutamate transport.

## Introduction

Alzheimer disease (AD), the main cause of dementia in old age, is characterized by β-amyloid deposition forming plaques and amyloid angiopathy, and hyperphosylated tau in neurons with neurofibrillary tangles (NFTs) and pre-tangles, dystrophic neurites of senile plaques, and neuropil threads (Duyckaerts and Dickson, 2011; Ferrer, 2012; Braak and Del Tredici, 2015). NFTs increase in number and distribution from stages I-II to stages V-VI with generalized involvement of the neocortex (Braak and Braak, 1991). Amyloid plaque burden also increases with disease progression from stages A (low density of plaques in the isocortex), B (medium density of plaques in the associative isocortex) and C (high density of plaques in motor and sensory isocortex) although there are important individual variations not related to NFTs (Braak and Braak, 1999). β-amyloid deposition has been categorized into phase 1 involving the neocortex, phase 2 with additional involvement of the allocortex, phase 3 involving the diencephalic nuclei, striatum, and cholinergic nuclei of the basal forebrain, phase 4 involving several nuclei of the brain stem, and phase 5 with cerebellar involvement (Thal et al., 2002).

Dementia with Lewy bodies (DLB), the second most common neurodegenerative dementia in the elderly, is pathologically characterized by Lewy bodies and Lewy neurites, composed of abnormal α-synuclein in the brainstem, limbic system and cortical areas (McKeith et al., 2004; Fujishiro et al., 2008; Ince, 2011).

Glutamate transporter solute carrier family 1, member 2 (GLT1/EAAT2) expressed in astrocytes regulates glutamate levels at the synapse and plays a cardinal role in preventing excitotoxic neuronal damage in certain neurodegenerative diseases (Nedergaard et al., 2002; Maragakis and Rothstein, 2006). Expression levels of GLT1 and glutamate homeostasis in prefrontal cortex in AD are controversial as they are reported to be decreased in some studies (Masliah et al., 1996) and preserved in others (Kulijewicz-Nawrot et al., 2013). An inverse relation between increased Glial fibrillary acidic protein (GFAP) and reduced GLT1 is described in AD with disease progression as defined by Braak stage of NFT pathology (Simpson et al., 2010, 2011). Indirect data point also to functional alterations of glutamate transporters in AD as a result of oxidative damage (Lauderback et al., 2001), altered solubility (Woltjer et al., 2010), and splice variants (Scott et al., 2011). Altered mRNA and/or protein expression of glutamate transporters have been reported in transgenic models of AD (Masliah et al., 2000; Cassano et al., 2012). Astrocytes in transgenic mice expressing mutant A53T α-synuclein have reduced expression of GLT1 (Gu et al., 2010) but there is no information, as far as we know, regarding GLT1 expression in DLB.

The present study analyses GLT1 mRNA and protein expression in frontal cortex in AD and DLB in a series of post-mortem human brains in order to learn about the possible implication of this astrocytic glutamate transporter in the pathogenesis of these diseases.

## Materials and Methods

### Human Cases

Brain tissue was obtained from the Institute of Neuropathology HUB-ICO-IDIBELL Biobank and the Hospital Clinic-IDIBAPS Biobank following the guidelines of Spanish legislation on this matter (Real Decreto de Biobancos 1716/2011) and approval of the local ethics committees. Processing of brain tissue has been detailed elsewhere (Ferrer, 2014). One hemisphere was immediately cut in coronal sections, 1 cm thick, and selected areas of the encephalon were rapidly dissected, frozen on metal plates over dry ice, placed in individual air-tight plastic bags, and stored at −80°C until use for biochemical studies. The other hemisphere was fixed by immersion in 4% buffered formalin for 3 weeks for neuropathological studies as detailed elsewhere (Ferrer, 2014). Neuropathological diagnosis of AD was categorized following Braak stages of NFT pathology adapted to paraffin sections (Braak and Braak, 1991; Braak et al., 2006) and Thal phases of β-amyloid plaques (Thal et al., 2002). DLB was diagnosed following accepted neuropathological criteria (Braak et al., 2003; McKeith et al., 2004; Alafuzoff et al., 2009). Middle-aged control cases (MA) had not suffered from neurological or psychiatric diseases, infections of the nervous system, brain neoplasms, systemic and central immune diseases, or metabolic diseases (including metabolic syndrome), and did not have abnormalities in the neuropathological examination excepting stages I–II of NFT pathology and phases 1–2 of β-amyloid plaques. Cases with associated pathologies such as vascular diseases (excepting mild atherosclerosis and arteriolosclerosis), TDP-43 proteinopathy, metabolic syndrome and hypoxia were excluded from the present study.

MA cases were 22 men and 17 women (*n* = 39, mean age 61.8 ± 14.0 years). AD cases, categorized as stages V–VI of NFT and phases 3–4 of β-amyloid, were 9 men and 11 women (*n* = 20, mean age 80.5 ± 6.9 years). DLB, categorized as stages 5–6 of Parkinson’s disease-related pathology, Thal phase 3–4, and NFT stages III–IV, were 8 men and 1 woman (*n* = 9, mean age 76.4 ± 5.7). The post-mortem delay was between 3 h and 9 h 35 min in MA, 2 h 30 min and 17 h 30 min in AD, and 5 h and 9 h in DLB cases.

### RNA Purification

Purification of RNA from right frontal cortex area 8 of human frozen brain was carried out using RNeasy Lipid Tissue Mini Kit (Qiagen, Hilden, Germany) following the protocol provided by the manufacturer combined with DNase digestion to avoid extraction and later amplification of genomic DNA. The concentration of each sample was obtained from A260 measurements with a NanoDrop 2000 spectrophotometer (Thermo Scientific, Waltham, MA, USA). RNA integrity was tested using the Agilent 2100 BioAnalyzer (Agilent, Santa Clara, CA, USA). Values of RNA integrity number (RIN) were between 6.4 and 8.4 in MA, between 6.4 and 9.1 in AD, and between 5.2 and 7 in DLB. Bivariate analyses were carried out to detect association of our variables with potential confounding factors (age, post-mortem delay and RIN) using Spearman or Pearson correlations for quantitative variables (data not shown). Statistical analysis was performed with GraphPad Prism version 5.01. post-mortem delay had no effect on RIN values in MA, AD and DLB cases.

### Retrotranscription Reaction

Retrotranscription reaction of RNA samples was carried out with the High-Capacity cDNA Archive kit (Applied Biosystems, Foster City, CA, USA) following the guidelines provided by the supplier, and using Gene Amp^®^ 9700 PCR System thermocycler (Applied Biosystems). A parallel reaction for one RNA sample was processed in the absence of reverse transcriptase to rule out DNA contamination.

### Real Time PCR

Real Time quantitative PCR (RT-qPCR) assays were conducted in duplicate on 1000 ng of cDNA samples obtained from the retro-transcription reaction, diluted 1:20 in 384-well optical plates (Kisker Biotech, Steinfurt, Germany) utilizing the ABI Prism 7900 HT Sequence Detection System (Applied Biosystems). Parallel amplification reactions were carried out using 20× TaqMan Gene Expression Assays and 2× TaqMan Universal PCR Master Mix (Applied Biosystems). Parallel assays for each sample were carried out using β-glucuronidase (*GUS-B*), X-prolyl aminopeptidase (aminopeptidase P) 1 (*XPNPEP1*) and alanyl-transfer RNA synthase (*AARS*) probes for normalization. The selection of these housekeeping genes was based on previous data showing low vulnerability in the brain of several human neurodegenerative diseases (Barrachina et al., 2006; Durrenberger et al., 2012). The reactions were performed using the following parameters: 50°C for 2 min, 95°C for 10 min, 40 cycles at 95°C for 15 s and 60°C for 1 min. TaqMan PCR data were captured using the Sequence Detection Software (SDS version 2.2, Applied Biosystems). Subsequently, threshold cycle (CT) data for each sample were analyzed with the double-delta CT (ΔΔCT) method. First, delta CT (ΔCT) values were calculated as the normalized CT values for each target gene in relation to the mean values of *GUS-β*, *XPNPEP1* and *AARS*. Second, ΔΔCT values were obtained with the ΔCT of each sample minus the mean ΔCT of the population of control samples (calibrator samples). The fold-change was determined using the equation 2^−ΔΔCT^. Genes analyzed with the corresponding abbreviations and TaqMan probes used in the study are shown in Table 1. Pearson’s correlation coefficient was used to assess a possible linear association between two continuous quantitative variables. To determine the relationship between gene expression and RIN values according to pathologic variables, we used the analysis of covariance. No significant correlations were found testing gender with diagnosis, post-mortem delay and RIN values. Taking into account that each group of pathological cases (AD and DLB) were processed in parallel with its respective group of controls, but not both pathologies in the same RT-qPCR plate, statistical *t*-test was chosen to compare sample groups instead of One-way ANOVA. Thus, statistical *t*-test was performed to analyze each pathological group with its respective control. Significant *p*-value: **p < 0.01.

**Table 1:** Taqman probes used in the present study including housekeeping genes (*GUS-B*, *XPNPEP1* and *AARS*), glutamate transporter 1 (*GLT1*), vesicular glutamate transporter 1 (*vGLUT1*), aldehyde dehydrogenase 1 family member 1 (*ALDH1L1*) and glial fibrillary acidic protein (*GFAP*).

Gene	Full name	Reference
**Housekeeping genes**		
*GUS-B*	β-glucuronidase	Hs00939627_m1
*XPNPEP1*	X-prolylaminopeptidase (aminopeptidase P) 1	Hs00958026_m1
*AARS*	Alanyl-tRNA synthetase	Hs00609836_m1
**Genes analyzed**		
*GLT1*	Glutamate transporter 1	Hs01102423_m1
*vGLUT1*	Vesicular glutamate transporter 1	Hs00220404_m1
*ALDH1L1*	Aldehyde dehydrogenase 1 family member 1	Hs01003842_m1
*GFAP*	Glial fibrillary acidic protein	Hs00909233_m1

### Gel Electrophoresis and Western Blotting From Total Homogenate

Tissue was processed as reported elsewhere (Garcia-Esparcia et al., 2015). A total of 0.1 g of frozen tissue from frontal cortex area 8 of all MA, AD and DLB cases was lysed with a glass homogenizer in Mila lysis buffer (0.5 M Tris at pH 7.4 containing 0.5 methylenediaminetetraacetic acid at pH 8.0, 5M NaCl, 0.5% Na deoxycholic acid, 0.5% Non-idet P-40, 1 mM phenylmethylsulfonyl fluoride, bi-distilled water, and protease and phosphatase inhibitor cocktails (Roche Molecular Systems, Pleasanton, CA, USA)), and then centrifuged for 15 min at 13,000 rpm at 4°C (Ultracentrifuge Beckman with 70Ti rotor, CA, USA). Protein concentration was measured with Smartspect™ plus spectrophotometer (Bio-Rad, CA, USA) using the Bradford method (Merck, Darmstadt, Germany). Samples containing 15 μg of protein and the standard Precision Plus Protein™ Dual Color (Bio-Rad) were loaded onto 10% acrylamide gels. Proteins were separated with sodium dodecylsulfate-polyacrylamide gel electrophoresis (SDS-PAGE) and electrophoretically transferred to nitrocellulose membranes using the Trans-BlotTurbo transfer system (Bio-Rad) at 200 mA/membrane for 60 min. Non-specific bindings were blocked by incubation with 5% milk in Tris-buffered saline (TBS) containing 0.1% Tween for 1 h at room temperature. After washing, the membranes were incubated at 4°C overnight with appropriate primary antibodies. Anti-GLT1 (Ab1783, Merck Millipore, Darmstadt, Germany) and anti-ALDH1L1 (ab56777, Abcam, Cambridge, UK) were used at a dilution of 1:500 in TBS containing 3% albumin and 0.1% Tween; both antibodies were detected after 1 h incubation with the appropriate HRP-conjugated secondary antibody (1:2000, Dako, Glostrup, Denmark), and the immune complexes were revealed with a chemiluminescence reagent (ECL, Amersham, GE Healthcare, Buckinghamshire, UK). Membranes were washed and incubated for 1 h with anti-β-actin antibody (1:30,000, A5316; Sigma-Aldrich, St. Louis, MO, USA), and blotted to control protein loading. After the immune complexes were revealed, membranes were stripped and blocked again by incubation with 5% milk in TBS containing 0.1% Tween for 1 h at room temperature. Once membranes were washed, they were incubated at 4°C overnight with rabbit anti-human GFAP (1:30,000, Z0334, Dako, Glostrup, Denmark) and detected after 1 h of incubation with anti-rabbit HRP-conjugated secondary antibody (1:2000, Dako, Glostrup, Denmark), and the immune complexes were revealed with ECL chemiluminescence reagent. Quantification of western blot optical densities (O.D.) was performed using *ImageJ* software (Bethesda, MD, USA), and analyzed with GraphPad Prism version 5.01 software (La Jolla, CA, USA) and Statgraphics Statistical Analysis and Data Visualization Software version 5.1 (Warrenton, VA, USA). Statistical *t-test* was performed to analyze each group with its respective control; significant *p-values* were established as: *p < 0.05 and **p < 0.01.

### Double-Labeling Immunofluorescence and Confocal Microscopy

For double-labeling immunofluorescence and confocal microscopy, de-waxed sections, 4 μm thick, of frontal cortex area 8 were stained with a saturated solution of Sudan black B (Merck Millipore, Darmstadt, Germany) to block the autofluorescence of lipofuscin granules present in cell bodies (15 min), and then rinsed in 70% ethanol and washed in distilled water. The sections were boiled in citrate buffer to enhance antigenicity and blocked for 30 min at room temperature with 10% fetal bovine serum diluted in PBS. The sections were incubated at 4°C overnight with combinations of primary antibodies GLT1 (1:500, Merck Millipore, Darmstadt, Germany), GFAP (1:400, Dako, Glostrup, Denmark) and β-amyloid (1:50, Sigma, St. Louis, MO, USA). After washing, the sections were incubated with Alexa488 or Alexa546 (1:400, Molecular Probes, Waltham, MA, USA) fluorescence secondary antibodies against the corresponding host species. Nuclei were stained with DRAQ5™ (1:2000, Biostatus, Leicestershire, UK). After washing, the sections were mounted in Immuno-Fluore mounting medium (ICN Biomedicals, Irvine, CA, USA), sealed, and dried overnight. Sections were examined with a Leica TCS-SL confocal microscope (Wetzlar, Germany).

## Results

### *GLT1*, *vGLUT1*, *ALDH1L1* and *GFAP* mRNA Expression in AD and DLB

**Figure 1:**
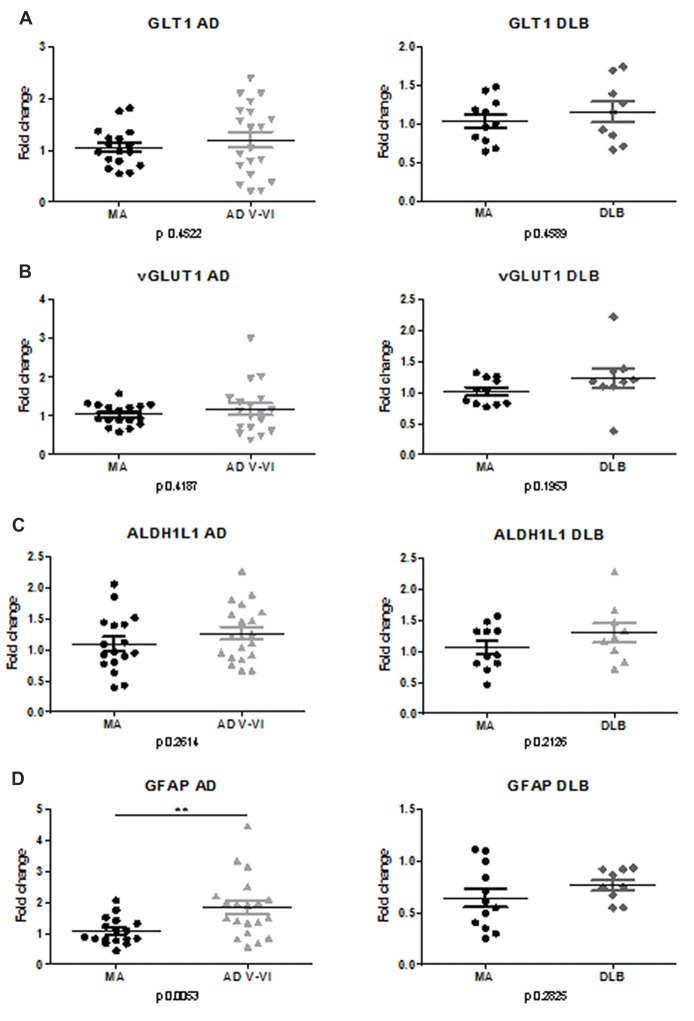
mRNA expression of *GLT1*, *vGLUT1*, Glial fibrillary acidic protein (GFAP*)* and *ALDH1L1* in frontal cortex area 8 in Alzheimer disease (AD) and dementia with Lewy bodies (DLB). Mean expression of housekeeping genes *GUS-B*, *XPNPEP1* and *AARS* was used to normalize samples. Variations are represented as Fold-change. No significant differences are observed regarding GLT1 **(A)**, *vGLUT1* **(B)**, *ALDH1L1* **(C)** expression in AD and DLB compared with middle-aged control cases (MA). **(D)** In contrast, significant increase in *GFAP* mRNA is found in AD but not in DLB. Statistical *t*-test was performed to compare each group. *p* values are depicted in every analysis. Significant *p*-value stated at **p < 0.01.

No differences in the expression levels of *GLT1* were detected in frontal cortex area 8 in AD and DLB when compared with MA cases (Figure 1A).

No differences in the expression levels of *vGLUT1* were detected in frontal cortex area 8 in AD and DLB when compared with MA (Figure 1B).

No differences in the expression of *ALDH1L1* were found in frontal cortex area 8 in AD and DLB compared with MA (Figure 1C).

Significant differences in GFAP mRNA expression (*p* < 0.01) were seen between AD and MA cases. Yet no differences were observed between DLB and MA (Figure 1D).

### GLT1, ALDH1L1 and GFAP Protein Levels in AD and DLB

**Figure 2:**
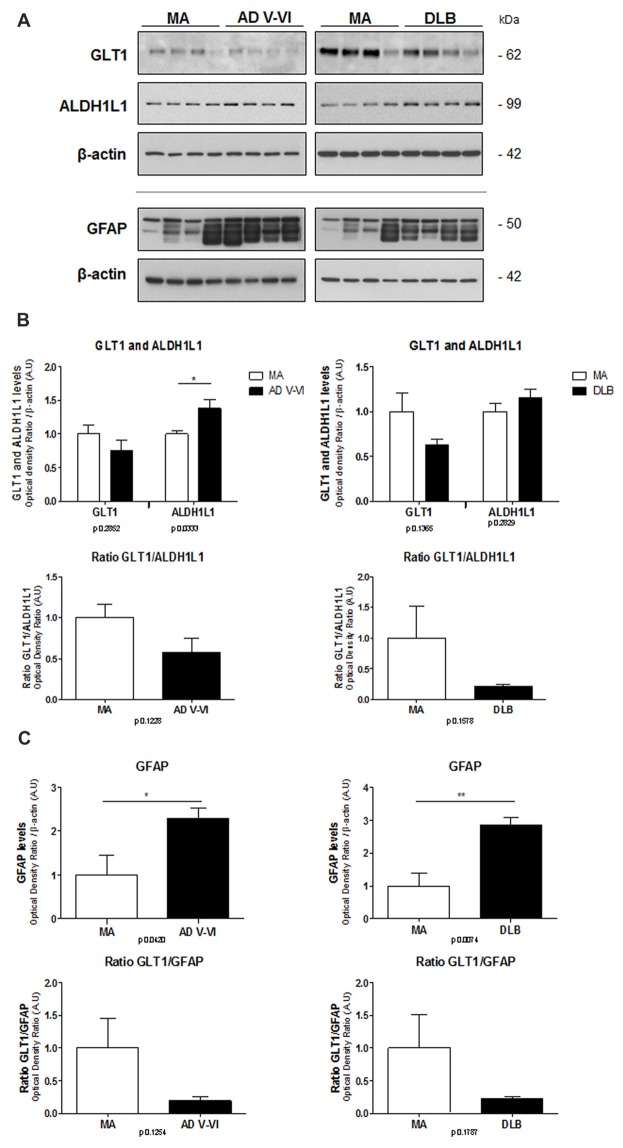
Protein expression levels of GLT1, ALDH1L1 and GFAP in frontal cortex in AD and DLB compared with MA. β-actin is used as control of protein loading **(A)**. No significant differences in the levels of GLT1 are found in AD and DLB compared with MA. However, significantly increased protein levels of ALDH1L1 are noted in AD, but not in DLB compared with MA. No significant differences, but just a trend to decrease, are found in the ratio between GLT1 and ALDH1L1 **(B)**. Significant increase in GFAP levels is found in AD and DLB when compared with MA cases. As a result, the ratio between GLT1 and GFAP is markedly reduced, although without statistical significance, in AD and DLB **(C)**. Statistical *t*-test: *p < 0.05, **p < 0.01. Ratios of optical densities between GLT1 and GFAP, and GLT1 and ALDH1L1 in MA are ascribed as value 1.

Protein levels of GLT1, ALDH1L1 and GFAP were assessed with gel electrophoresis and western blotting using β-actin as control of protein loading (Figure 2A).

GLT1 protein levels showed a tendency to decrease in AD and DLB without statistical significance. In contrast, ALDH1L1 protein levels were significantly increased in AD (*p* < 0.05) but not in DLB. As a result, the ratio between GLT1 and ALDH1L1 showed a trend to decrease without statistical significance in AD and DLB (Figure 2B).

GFAP protein levels were significantly increased in AD and DLB (*p* < 0.05 and *p* < 0.01, respectively). The ratio between optical densities of GLT1 and GFAP in MA cases was considered as ratio 1. The ratio between optical densities of GLT1 and GFAP showed a non-significant decrease in AD and DLB when compared with MA (Figure 2C).

### Double-Labeling Immunofluorescence and Confocal Microscopy

**Figure 3:**
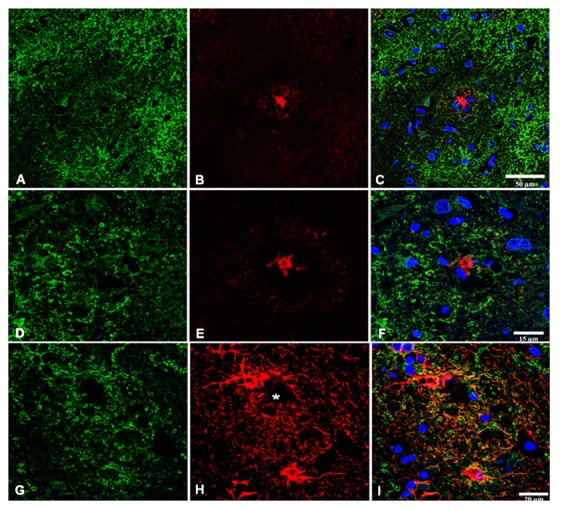
Double-labeling immunofluorescence and confocal microscopy of GLT1 (green), β-amyloid (red; **B,C,E,F**), and GFAP (red; **H,I**) in frontal cortex area 8 in AD. GLT1 immunoreactivity is preserved in the vicinity of β-amyloid plaques **(A–F)**. Astrocytes surrounding small β-amyloid deposit (asterisk) show preserved GLT1 immunoreactivity at the cell membrane **(G–I)**. Paraffin sections, nuclei (blue) are stained with DRAQ5™; **(A–C)**, bar = 50 μm; **(D–F)**, bar = 15 μm; **(G–I)**, bar = 20 μm.

Double-labeling immunofluorescence and confocal microscopy disclosed preservation of GLT1 immunofluorescence surrounding β-amyloid plaques (Figures 3A–F). At higher magnification, astrocytes showed GLT1 immunofluorescence at the cell membrane (Figures 3G–I).

## Discussion

Present observations show preserved *GLT1* mRNA expression in cerebral cortex area 8 in AD and when compared with MA individuals. A tendency of GLT1 protein levels to decrease, with no statistical significance, is found by western blotting in AD and DLB. Moreover, GLT1 immunoreactivity localizes equally in reactive astrocytes in the vicinity of β-amyloid plaques and in astrocytes with no apparent association with plaques. This would suggest no modifications of GLT1 expression in AD. In contrast, significant increase of GFAP mRNA and protein expression occurs in AD and DLB. Therefore, GLT1 protein levels do not parallel GFAP protein levels in frontal cortex in AD and DLB. This observation is in line with previous reports indicating an inverse relation between increased GFAP expression and reduced GLT1 expression with disease progression in AD (Simpson et al., 2010, 2011). This is further demonstrated by analyzing the ratio between GLT1 and GFAP protein levels. A decreased ratio, although not significant, occurs in AD and DLB, thus suggesting an imbalance between GFAP and GLT1 expression in astrocytes in these diseases. However, this interpretation must be taken with caution as not all astrocytes express GFAP and other astrocyte markers may not be expressed in GFAP-immunoreactive astrocytes (Kimmelberg, 2004; Sofroniew and Vinters, 2010; Oberheim et al., 2012).

ALDH1L1 has been used as a marker of total astrocytes (Ferrer, 2017). No significant differences in *ALDH1L1* mRNA expression are seen in AD and DLB when compared with MA. However, significant increase in ALDH1L1 protein levels is found in AD but not in DLB, thus indicating an increase in the number of astrocytes in AD when compared with MA. The ratio between ALDH1L1 and GLT1 is decreased, although not significantly, in AD and DLB, thus suggesting an imbalance between the total number of astrocytes and GLT1 protein levels.

These subtle differences do not necessarily indicate primary alterations in the production of GLT1 in astrocytes, but rather secondary adaptations to neuronal dysfunction. GLT1 expression depends on the number of functional synapses under physiological and pathological conditions (Kugler and Schleyer, 2004; Genoud et al., 2006; Yang et al., 2009). Since synaptic connectivity is reduced in frontal cortex in AD and DLB (Jellinger, 2000; Selkoe, 2002; Hashimoto and Masliah, 2003; Gong and Lippa, 2010; Schulz-Schaeffer, 2010; Ferrer, 2012; de Wilde et al., 2016; Santpere et al., 2017), the possibility of reduced GLT1 expression secondary to decreased synaptic activity must not be overlooked in AD and DLB.

## Author Contributions

PG-E and DD-L carried out biochemical study of AD and DLB. MA, BT-E and MC performed immunohistochemistry. FL helped in the design of the study. IF selected the cases, designed and supervised the study and wrote the manuscript, which was circulated among the authors for approval.

## Conflict of Interest Statement

The authors declare that the research was conducted in the absence of any commercial or financial relationships that could be construed as a potential conflict of interest.
